# Epigenetics knocks on synthetic biology's door

**DOI:** 10.1093/femsle/fnw191

**Published:** 2016-08-11

**Authors:** Zuemy Rodriguez-Escamilla, Mario A. Martínez-Núñez, Enrique Merino

**Affiliations:** 1 Departamento de Microbiología Molecular, Instituto de Biotecnología, UNAM. Av. Universidad 2001, Cuernavaca, Morelos CP 62210, México; 2 Laboratorio de Ecogenómica. Unidad Académica de Ciencias y Tecnología de Yucatán. Facultad de Ciencias, UNAM. Sierra Papacal-Chuburna Km 5. Mérida, Yucatán CP 97302, México

**Keywords:** epigenetic circuits, heritable cellular fates, hysteresis, T7 RNA polymerase, Sigma 70, Excludon

## Abstract

Epigenetics is the study of heritable changes in gene expression without concomitant changes in DNA sequence. Due to its relevance in development, differentiation and human health, epigenetics has recently become an emerging area of science with regard to eukaryotic organisms and has shown enormous potential in synthetic biology. However, significant examples of epigenetic regulation in bacterial synthetic biology have not yet been reported. In the current study, we present the first model of such an epigenetic circuit. We termed the circuit the *alternator circuit* because parental cells carrying this circuit and their progeny *alternate* between distinct and heritable cellular fates without undergoing changes in genome sequence. Furthermore, we demonstrated that the *alternator circuit* exhibits hysteresis because its output depends not only on its present state but also on its previous states.

## Abstract (precis)

First example of bacterial regulatory circuit with epigenetic traits created by Synthetic Biology.

## INTRODUCTION

Designing, modeling and generating complex biological systems with novel metabolic or cellular phenotypes is the essence of synthetic biology. The development of new models in this emerging area has offered unprecedented opportunities to enhance understanding of the biological processes present in living organisms. These opportunities far exceed original expectations and have shown real promise for transforming biotechnology and medicine. Since its beginnings in the early 1990s, key breakthroughs in synthetic biology have resulted from the incorporation of basic elements and concepts from molecular and cellular biology, such as the design of regulatory circuits based on transcription factors [e.g. toggle switches (Gardner, Cantor and Collins 2000) and the repressilator (Elowitz and Leibler 2000)], riboregulators (Isaacs *et al.*^2004^), antisense small RNA, CRISPR-mediated transcription silencing (Jiang *et al.*^2013^) and quorum-sensing genes for cell–cell communication to enable multicellular patterning (Basu *et al.*^2005^), among others. The current study utilizes, for the first time, the concept of epigenetic regulation (Burggren and Crews 2014) to design and construct a bacterial regulatory circuit.

Epigenetic-mediated gene expression is almost exclusively found in eukaryotic organisms and primarily involves changes in DNA methylation or histone modification. Epigenetic processes have central roles in numerous cellular processes, including embryonic development, X-chromosome inactivation, genomic imprinting and maintenance of chromosomal stability (reviewed in Goldberg, Allis and Bernstein 2007). In bacterial organisms, DNA methylation-mediated gene regulation has been shown to play an important role in coordinating different cellular processes, such as sDNA replication, chromosomal segregation, mismatch repair and gene transcription regulation associated with virulence and pathogenesis (reviewed in Casadesús and Low 2006). In some cases, hemimethylated or non-methylated DNA patterns can be inherited by daughter cells, reflecting the metabolic state of a cell prior to its undergoing division and providing future generations with enhanced abilities to adapt to growth conditions. Whether such regulation by bacterial DNA methylation can be considered epigenetic regulation remains controversial because it is only present in the short term and is not a stable, heritable phenotype (Zhu *et al.*^2016^).

To the best of our knowledge, there are no documented examples of regulatory processes based on epigenetic mechanisms in bacterial synthetic biology. Thus, the development of such regulatory circuits could provide unprecedented opportunities for the construction of new and better types of synthetic systems. As a first example of the design of a control circuit based on epigenetic traits in bacteria, we have created a regulatory circuit that we termed the *alternator circuit* because it allows the bacteria carrying it to alternate between different cellular states.

## MATERIALS AND METHODS

### Plasmids and strain construction

**Figure 1.**
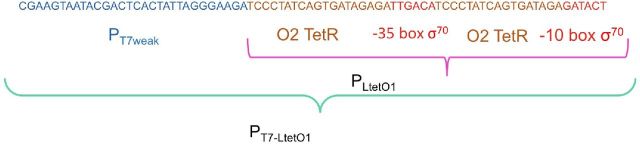
Construction of the *P_T7-LtetO1_* promoter region. The colors of the nucleotide sequence correspond to the following molecular elements: Blue: P_T7weak_ promoter; brown TetR operator O2 and red: –35 and –10 σ^70^ promoter boxes. The DNA fragment containing this regulatory region was obtained by PCR amplification.

**Figure 2.**
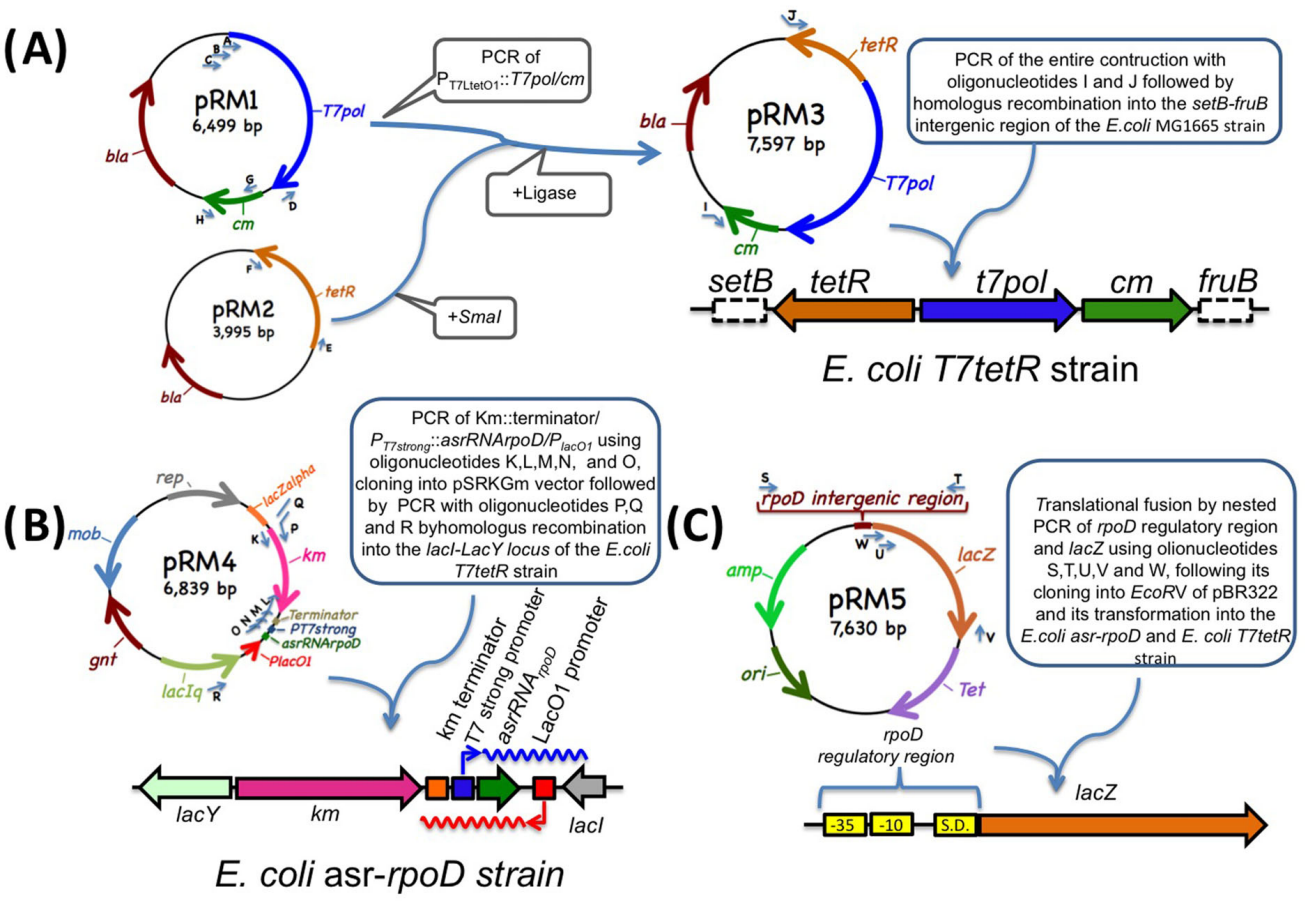
Genetic engineering required for the construction of strains and plasmid used in our study. (**A**) Construction of the *E. coli T7tetR* strain (*setB/tetR/P_T7-LtetO1_::T7pol/cm/fruB*). This strain was used as a receptor strain for posterior integrations of the *asrRNA* gene. (**B**) Construction of the *E. coli asr-rpoD* strain (*setB/tetR/P_T7-LtetO1_::T7pol/cm/fruB/lacY/Km::terminator/P_T7strong_::asrRNArpoD::P_lacO1_/lacI*). (C) Construction of the pRM5 plasmid. This plasmid carries the *rpoD-lacZ* translational fusion cloned into the *EcoR*V restriction site of the plasmid pBR322.

Three different *Escherichia coli* strains were constructed. The first was the *E. coli T7tetR* (*setB/tetR*/*P_T7-LtetO1_*::*T7pol*/*cm*/*fruB*) (see Figs 1 and 2A), which was used as a receptor strain for the integration of the *asrRNArpoD* gene between the *lacI* and *lacY* genes, generating the *E. coli* asr-*rpoD* strain (*setB/tetR/P_T7-LtetO1_::T7pol/cm/fruB/lacY/Km::terminator/P_T7strong_::asrRNArpoD::P_lacO1_/lacI*) (Fig. 2B).

The *E. coli T7tetR* strain was constructed using the following procedure: (1) PCR amplification of genes and specific intergenic regions using AccuPrime *Pfx* Polymerase (Invitrogen). To obtain the *P_T7-LtetO1_*::*T7pol* fusion gene (Fig. 1), we used oligonucleotides A, B, C and D, shown in Table 1, and *E. coli BL21* chromosomal DNA as a template. To amplify the *tetR* gene, we used the oligonucleotides E and F and *E. coli XL1 blue* chromosomal DNA as a template. To amplify the *cm* gene, we used the oligonucleotides G and H and pBR325 DNA as a template. (2) The PCR-amplified fragments were cloned into a pMOS blue vector (Amersham) and subsequently amplified using oligonucleotides I and J, which carried extra 50 bp long fragments with sequences identical to the intergenic regions of *setB* and *fruB*, respectively, for subsequent homologous recombination. (3) The final construct was integrated into the *E. coli* MG1665 genome by homologous recombination using the λ red system (Datsenko and Wanner 2000).

**Table 1.** Oligonucleotides used for amplification of DNA. The letter ID, name and 5′–3′nucleotide sequence of the oligonucleotides used for the PCR DNA amplifications used in our study are indicated.

ID	Name	Sequence
A	tetO-t7	CTA TCA GTG ATA GAG ATA CTG AGC ACA TCA AGA GGC ACT AAA TGA ACA CGA TTA AC
B	A-tetO	ATC CCT ATC AGT GAT AGA GAT TGA CAT CCC TAT CAG TGA TAG AGA TAC TGA GCA CAT C
C	pT7-weak-ptetO1	CGA AGT AAT ACG ACT CAC TAT TAG GGA AGA TCC CTA TCA GTG ATA GAG ATT GAC
D	polt7-3′	TTA CGC GAA CGC GAA GTC CGA C
E	tetR-interg-5′	CCA ACC GAA CCA CTT CAC GCG T
F	tetR-3′	GCT TTT AAG ACC CAC TTT CAC ATT TAA G
G	cm-5′	GAG ACG TTG ATC GGC ACG TAA G
H	cm-3′	AGC ACA CGG TCA CAC TGC TTC C
I	fruB	GAT CGC GCT GAA ACG TTT CAA GAA AGC ATA ATA CTT CTG TTT CAG CAC GC
J	setB	CAG CAA TTA GGA AAA ATG GCA AAA AAT TGT GCA GCA CAT CAA ACT TTT GCT C
K	km-5′	GGA CAG CAA GCG AAC CGG AAT TG
L	km-Terminator	AAA AAC GCA TCA ATC AAA TTG ATG CGG AAT CGA AAT CTC GTG ATG GCA GG
M	Term-pT7strong	GTT GTG GTC TCC CTA TAG TGA GTC GTA TTA AAA ACG CAT CAA TCA AAT TGA TGC
N	arRNArpoDpt7stro	CCC TCA TGA AAT AAG TGT GGA TAC CGT TGT GGT CTC CCT ATA GTG AG
O	arRNArpoD	AAA AAG CGC GAA AAA CGC GCC CTC ATG AAA TAA GTG TGG ATA CC
P	km-lacY	CAA AAA TAA TAC CCG TAT CAC TTT TGC GGA CAG CAA GCG AAC CGG AAT TG
Q	lacY	GCG AGA ACA GAG AAA TAG CGG CAA AAA TAA TAC CCG TAT CAC TTT TGC
R	lacI	GGG CGC AAT GCG CGC CAT TAC C
S	rpoD-intergenic-5′	TTT AAC GGC TTA AGT GCC GAA GAG
T	rpoD-intergenic-3′	AAG ACG GTA TCC ACA CTT ATT TCA TG
U	lacZ-5′	ATG ACC ATG ATT ACG GAT TCA CTG G
V	lacZ-3′	TTA TTT TTG ACA CCA GAC CAA CTG G
W	rpoD-lacZ-fusion	CAT GAA ATA AGT GTG GAT ACC GTC TTA TGA CCA TGA TTA CGG ATT CAC TGG

The *E. coli* asr-*rpoD* strain was constructed using the following procedure: (1) The *Km* gene was amplified using oligonucleotides K and L and pKD4 DNA as a template. This gene was chosen as a selection marker for homologous recombination to integrate the *P_T7strong_::asrRNArpoD locus* into the *E. coli T7tetR* receptor strain between the *lacI* and *lacY* genes of the *E. coli* MG1665 strain (Fig. 2A). (2) The *Km* gene was fused to the *P_T7strong_::arsRNArpoD locus* by PCR amplification using the K-M-N-O oligonucleotides. (3) Subsequently, the PCR-amplified product was cloned into the *Sma*I site of the pSRKGm plasmid (Khan *et al.*^2008^) to generate the pRM4 plasmid. (4) The plasmid pRM4 was subsequently used as template for a PCR amplification of the *Km::terminator/P_T7strong_::asrRNArpoD::P_lacO1_ locus* (see Fig. 2B) using the P, Q and R oligonucleotides (see Table 1). The resulting DNA amplified fragment is flanked by *lacY* and *lacI* sequences required for a posterior homologous recombination in the recipient *E. coli T7tetR* strain using the λ red system (Datsenko and Wanner 2000) to generate the *E. coli* asr-*rpoD* strain (Fig. 2B).

To evaluate the inhibition efficiency of the *asrRNArpoD* gene on *rpoD* translation, a *rpoD*-*lacZ* translational fusion was constructed using the following procedure: (a) The *rpoD* regulatory region and the *lacZ* gene were PCR amplified from the *E. coli* MG1665 strain using the S-T and U-V oligonucleotides, respectively (see Table 1). (b) These two fragments were used as templates and fused by a PCR amplification using the S, V and W oligonucleotides and subsequently cloned into the pBR322 *EcoR*V site. The resulting plasmid was the pRM5 (see Fig. 2C). This plasmid was used to transform the *E. coli T7tetR* and *E. coli asr-rpoD* strains to obtain their corresponding derivatives, the *E. coli T7tetR/*pRM5 and *E. coli asr-rpoD/*pRM5 strains, respectively.

### Growth cultures

The *E. coli T7tetR, E. coli asr-rpoD* and their derivative strains transformed with the pRM5 plasmid were grown in 10 mL of LB liquid medium overnight (ON). Subsequently, 5 μL of the ON cultures were used to inoculate 280 μL of fresh LB medium with and without the addition of anhydrous tetracycline (ATc) to a final concentration of 100 ng mL^−1^. These cultures were grown for 13 h, washed with an isotonic saline solution and then used to inoculate a second set of cultures that were grown in LB medium without ATc. The second set of cultures was also grown for 13 h. Changes in optical density or fluorescence were assessed in triplicates. Measurements were performed every 20 min in an ELISA plate reader (Synergy BioTek). The β-galactosidase activities of the different strains were measured by the hydrolysis of fluorescein di-D-galactopyranoside that was added to a final concentration of 50 ng mL^−1^. A *P_lacO1_* promoter was introduced downstream and in the complementary strand of the *asrRNArpoD* gene (see Fig. 2). The transcription induction from this *P_lacO1_* promoter was obtained by the addition of Isopropyl β-D-1-thiogalactopyranoside (IPTG) to a final concentration of 25 μg mL^−1^.

### Statistical analysis

We used a nested sampling Bayesian approach to compare bacterial growth cultures as described by Rickett *et al.* (2015). The Bayes’ factor (B13) was calculated using the four-parameter Baranyi model (Baranyi, Roberts and McClure 1993) to test hypothesis 1 (H1), i.e. ‘data curves are replicates’, versus hypothesis 3 (H3), i.e. ‘all data curve parameters are different’, in the *babar* R package. Jeffreys’ scale was use to interpret the Bayes’ factor by calculating 2ln (B13) for H1 versus H3.

## RESULTS AND DISCUSSION

**Figure 3.**
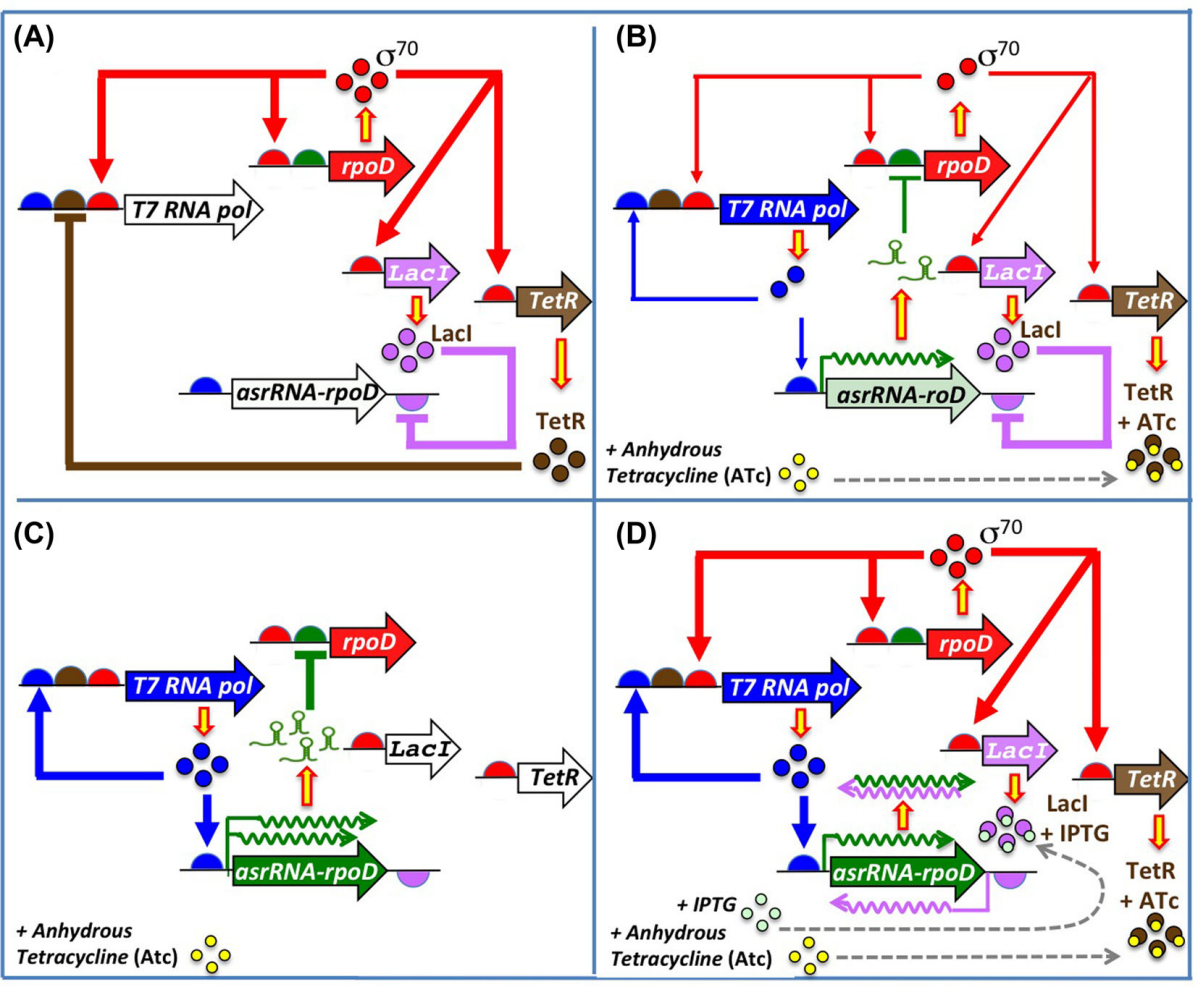
The alternator circuit. (**A**) *Circuit at baseline*. TetR is attached to the *T7pol 5*′ regulatory region, blocking its transcription. (**B**) *Circuit after induction and transition state*. After ATc is added to the medium, it interacts with TetR, triggering a conformational change to a non-repressible form. Following this, the *tetR* operator region is released, and transcription of the *T7pol* gene occurs via the σ^70^-RNAP holoenzyme, T7pol begins to accumulate in the cytoplasm and direct the transcription of both the *asrRNArpoD* gene and of its own gene. As result of this transcription, the translation of *rpoD* mRNA starts to be blocked by asrRNArpoD, and the intracellular concentration of σ^70^ starts to decrease. (**C**) Final stage of the circuit. T7 RNAP and σ^70^ reach their maximum and minimum intracellular levels, respectively. The decrease in σ^70^ concentration leads alternative sigma factors, such as σ32, to interact with RNAP to form active RNAP-σ complexes, resulting in an alternative transcription pattern. The feedback loop of the circuit remains stable. (**D**) *Reversion of the epigenetic traits.* The addition of IPTG to the medium induces the transcription of long antisense asrRNArpoD from an inducible P_LacO1_ promoter located at the 3′end of this locus and in the complementary strand. These long antisense RNAs bind the asrRNArpoD transcripts and block their inhibitory activity on the *rpoD* translation. Consequently, the epigenetic traits of the strain are reversed.

Our *alternator circuit* is comprised of the following elements, which are shown in Fig. 3.

1. The RNA polymerase (RNAP) gene from bacteriophage T7 (T7pol). Due to the interactions of the elements involved in our regulatory circuit, *T7pol* gene transcription depends on both the P_LtetO1_ (Lutz and Bujard 1997) and the *P_T7weak_* (bacteriophage T7—parts.igem.org) promoters, the transcription of which depends on σ^70^ and T7pol, respectively (see the Materials and methods section). There are two TetR operators in the synthetic regulatory region of this gene: one located between and the other located in front of the P_LtetO1_ and P_T7weak_ promoters (for details, see Fig. 1 and the Materials and methods section). This configuration ensures that *T7pol* gene transcription in the context of the circuit is under the control of TetR, regardless of which promoter transcribes the gene.
2. The wild-type *rpoD* gene, which encodes the housekeeping factor σ^70^.
3. An **a**rtificial **s**mall **r**egulatory RNA (asrRNA) gene designed to bind to a region within *rpoD* mRNA that overlaps with the Shine–Dalgarno sequence, thus inhibiting the synthesis of σ^70^. Due to the nature of its regulatory target, the transcription of the *asrRNArpoD* gene was designed to depend on the *T7pol* promoter (P_T7strong_ (bacteriophage T7—parts.igem.org)) and not on a *bonafide* σ^70^ promoter. This construction was integrated between the *lacI* and *lacY* genes of the *Escherichia coli* MG1665 strain by double homologous recombination. The resultant strain was designated *E. coli* asr-*rpoD* (for details, see Fig. 2 and the Materials and methods section).
4. In this strain, immediately downstream and in the complementary strand of this *asrRNArpoD* gene, we have integrated a *P_lacO1_* promoter (see Fig. 2). In the presence of the gratuitous inducer IPTG, long asrRNArpoD antisense RNAs are transcribed and block the regulatory activity of the *asrRNArpoD* sense RNAs. This kind of long antisense RNAs that spans divergent genes or operons with opposing functions has been termed *Excludon* (Sesto *et al.*^2012^).
5. A gene encoding the TetR regulator that required a constitutive promoter recognized by the RNAP-σ^70^ holoenzyme of *E. coli* for successful transcription.

### Our experimental procedure involved two consecutive growth conditions

(1) *Initial bacterial culture in Luria–Bertani (LB) broth*. Our *E. coli* asr-*rpoD* and its parental *E. coli T7tetR* strain were initially grown in two different media. The first was composed of LB broth without ATc, and the second, which was used for induction, was composed of LB broth with the addition of ATc (for details, see the Materials and methods section).

**Figure 4.**
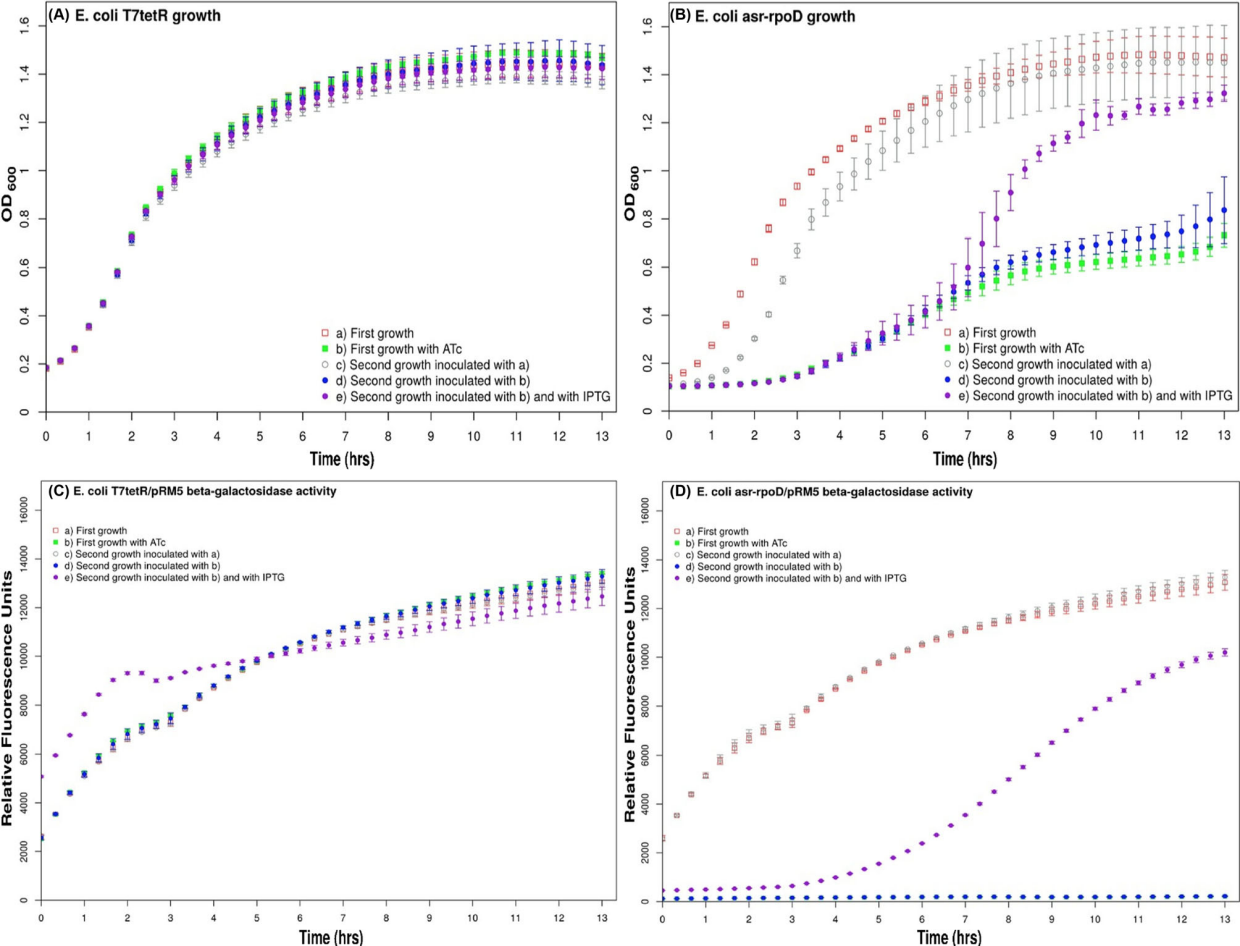
Impact of the epigenetic regulation of *σ70* on *E. coli* growth rate and on the β-galactosidase activity of a *E. coli* strain carrying an *rpoD-lacZ* translation fusion. (**A**) The *E. coli T7tetR* strain and (**B**) its derivative *E. coli asr-rpoD* strain were growth in LB medium in the absence (empty shapes) and presence (filled shapes) of ATc as a TetR inducer. An initial set of cultures (squares) was used to inoculate a second set of cultures (circles). Note that the growth rates of the *E. coli ars-rpoD* strains depend on the growth conditions of their inoculum, which indicates an epigenetic trend. The *E. coli T7tetR* strain used as a control was subjected to the same conditions as above. Nevertheless, in this case, no epigenetic regulation was observed. The *E. coli T7tetR* and *E. coli asr-rpoD* strains were transformed with the pRM5 plasmid that carries an *rpoD-lacZ* translation fusion as a reporter gene. The same growth procedure was carried out with these strains to analyze the epigenetic regulatory activity of our asrRNArpoD on the translation of the *rpoDlacZ* reporter gene measured by the β-galactosidase activities of the strains. (**C**) No epigenetic trait is observed in the *E. coli T7tetR/*pRM5 strain. (**D**) A clear epigenetic trait is observed in our *E. coli asrrpoD*/pRM5 strain. This epigenetic trait is reversed by the addition of IPTG to the media that induces the transcription of a long antisense asrRNArpoD from a *P_lacO1_* promoter.

In the LB medium without ATc, TetR repressed the transcription of the *T7pol* gene. As such, T7pol was not available to transcribe the *asrRNArpoD* gene, and *rpoD* mRNA was fully translated (Fig. 3A). As a result, the growth rate of this strain was similar to that of the *E. coli T7tetR* control strain (Fig. 4A and B).

In the LB induction medium with ATc, the TetR-mediated repression of *T7pol* gene transcription was eliminated due to circuit activation. Therefore, the *asrRNArpoD* gene was transcribed, and the resultant RNA product blocked the translation of σ^70^ mRNA (Fig. 3B and C). As the intracellular concentration of σ^70^ decreases, other sigma factors may form transcriptional complexes with the RNAP core, altering the transcriptional pattern of the bacteria and leading to a concomitant reduction in growth rate (Fig. 4B).

(2) *Secondary bacterial culture in LB medium without ATc.* Two *E. coli asr-rpoD* cell cultures were grown in non-induction LB media. The first culture was inoculated with a culture never exposed to induction medium, and the second culture was inoculated with a culture previously exposed to induction medium. As shown in Fig. 4A and B and based on statistical analysis (for details, see the Materials and methods section), the second cultures presented different growth rates, despite that they were grown in the same non-induction media and possessed identical genotypes. A growth rate close to 0.1 OD_600_/h was observed in all of the *T7tetR* strains regardless the growth conditions of their inoculum, and also in the *E. coli* asr-*rpoD* strains whose inoculum were not exposed to ATc, were close to 0.1 OD_600_/h; on the other hand, a much more smaller growth rate of 0.03 OD_600_/h was observed in the *E. coli* asr-*rpoD* strain whose inoculum was exposed to ATc. Therefore, these results clearly demonstrate the inheritance of an epigenetic trait. Furthermore, because the alternative outputs (i.e. growth rates) of these cultures showed time-based dependence on both present (i.e. growth conditions of the culture) and past (i.e. growth conditions of the inocula) inputs, our regulatory circuit exhibited hysteresis.

In order to demonstrate that the observed reduction growth rates epigenetically inherited to the offspring generations was due to the translation inhibition of *rpoD* by our *asrRNArpoD* gene; the initial and secondary bacterial cultures described above were repeated. In this second occasion, the *E. coli T7tetR* and *E. coli* asr-*rpoD* strains were transformed with the pRM5 plasmid. This plasmid carries an *rpoD-lacZ* translational fusion as a reporter gene (see Fig. 2C). It is important to note the *E. coli asr-rpoD* strain does not have detectable β-galactosidase activities since their corresponding chromosomal *lacZ* genes were deleted by the integration of the *Km::terminator/P_T7strong_::asrRNArpoD::P_lacO1_ locus* the *lacI* and *lacY* genes. As it can be seen in Fig. 4, the epigenetic trait observed as differences in the growth rates of the *E. coli asr-rpoD* strains can also be clearly observed as a drastic change in the β-galactosidase activity of the *E. coli asr-rpoD*/pRM5 strain when inoculated with differently induced cultures.

As a second approach to demonstrate that the epigenetic traits observed in our *E. coli asr-rpoD* strain were due to the translation inhibition of *rpoD*, we included an inducible LacO1 promoter at the 3′ end of the *asrRNArpoD locus*. This promoter is located in the complementary strand, therefore, its induction by the addition of IPTG to the medium results in the transcription of a long antisense asrRNArpoD that binds and blocks the regulatory effect of the asrRNArpoD on the *rpoD* translation. In Fig. 4B and D, it can be seen that the IPTG induction of the antisense asrRNArpoD reverts the epigenetically acquired phenotypes of the *E. coli asr-rpoD* strains reflected in their growth rate (from 0.03 OD_600_/h to 0.1 OD_600_/h) and β-galactosidase activity of our *rpoD-lacZ* translational fusion.

We selected *rpoD* as the main regulatory target of our epigenetic system due to its important role in cellular metabolism. Our aim was to illustrate how epigenetic circuits created in synthetic biology can generate alternative, distinctive and heritable cellular fates without the need to alter DNA genome sequences. In *E. coli*, there are seven sigma factors, including the housekeeping σ^70^ as well as six alternative sigma factors that are required under specific physiological conditions. Among the different sigma factors, σ^70^ is maintained at the highest intracellular concentration and possesses the highest affinity for RNAP (Maeda, Fujita and Ishihama 2000); therefore, it is unsurprising that σ^70^ regulates at least 50% of all *E. coli* genes (Thieffry *et al.*^1998^). However, in certain metabolic conditions, the relative concentration of σ^70^ with respect to the concentrations of the alternative sigma factors may decrease, favoring the replacement of σ^70^ in RNAP (Sharma and Chatterji 2010). In our epigenetic system, we altered the intracellular concentration of σ^70^ by artificial inhibition of *rpoD* translation. This resulted in the replacement of σ^70^ in RNAP with alternative sigma factors, generating stress and concomitantly reducing cell growth rate. A similar phenotype of arrested growth rate has been observed in the *rpoD* knockout *E. coli* strain reported by Keio (Baba *et al.*^2006^). However, it is notable that the slow growth rate phenotype is associated with DNA modification in this case (i.e. the deletion of the chromosomal *rpoD* gene); therefore, all descendant cells would exhibit the same phenotype. On the contrary, epigenetically governed traits can alternate between phenotypes without new genotype alterations. For example, in the current study, phenotypic alterations were induced simply by changing the intracellular concentration of σ^70^.

Unlike canonical epigenetic processes, our synthetic biology model does not depend on DNA methylation or histone modifications. Rather, this model relies on the establishment of a regulatory feedback loop that begins with the transcriptional activation of the T7 RNAP gene. To illustrate the significant phenotypic changes that can be obtained using this epigenetic system, our design included translation regulation of the housekeeping σ^70^ factor via the use of arsRNA. This set-up results in clear changes in metabolic responses. Although our *regulator circuit* was developed for basic research purposes, the current work establishes the principles needed to develop novel regulatory circuits that are under epigenetic control and exhibit hysteresis. Such a design can be applied to produce changes in the transcriptional patterns of organisms to promote the overproduction of specific biological products. It will be interesting to witness the evolution of epigenetic circuits in synthetic biology in the future.
